# Influence of Atomic Magnetometer’s Orientation on Its Frequency Response

**DOI:** 10.3390/s25051364

**Published:** 2025-02-23

**Authors:** Rui Zhang

**Affiliations:** National Innovation Institute of Defense Technology, AMS, Beijing 100071, China; dr.ruizhang@163.com

**Keywords:** atomic magnetometer, frequency response, orientation

## Abstract

Due to the high sensitivity and room temperature operation of atomic magnetometers, they have significant applications in many fields. An emerging area is the highly sensitive biomagnetic measurement in magnetically unshielded environments, which is crucial for medical diagnostics. However, in magnetically unshielded environments, atomic magnetometers often encounter situations where their orientation deviates from the optimal operating posture, and there has been insufficient research on the frequency response information of atomic magnetometers under such conditions. Addressing this issue, we modeled the atomic magnetometer using the Bloch equations and obtained approximate analytical solutions for the frequency response of the atomic magnetometer in different orientations, which were experimentally verified using a Bell–Bloom magnetometer. We found that although the magnetic resonance spectrum of the magnetometer is influenced by the orientation of the magnetometer, the frequency response of the magnetometer can be made independent of its orientation by judiciously selecting the demodulation components used for the operation of the magnetometer. This finding is important for biomagnetic detection in magnetically unshielded environments where orientation-robust measurements of AC magnetic signals are required.

## 1. Introduction

Portable atomic magnetometers [1,2,3,4], which combine high sensitivity, compact size, and cryogen-free operation, have found extensive applications in magnetically unshielded environment, such as geophysical exploration and related fields [5,6,7,8,9]. Breakthroughs in sensitivity enhancement and miniaturization [1,10,11,12,13,14,15,16,17,18] have further established atomic magnetometers as viable tools for biomagnetic sensing in magnetically shielded environments [19,20,21] while demonstrating emerging potential for biomagnetic detection in unshielded settings [22].

However, in these unshielded environmental applications, magnetometers are inevitably subjected to orientation variations relative to the background magnetic field. This consequently results in significant orientation-dependent performance variations in atomic magnetometers. This directional dependence originates from their operational principle: atomic magnetometers measure magnetic fields by detecting the Larmor precession of optically polarized atomic spins. Given that both the optical pumping axis and signal detection axis are geometrically fixed within the instrument’s configuration, any reorientation of the device relative to the ambient magnetic field modifies the spin precession dynamics, thereby inducing anisotropy in critical performance parameters including sensitivity, measurement accuracy, and frequency response. Such orientation-dependent characteristics impose significant constraints on practical deployments in mobile platforms or complex magnetic environments.

Previous studies on the orientation effects of atomic magnetometers have focused predominantly on orientation-induced dead zones and heading errors [1]. The dead zone phenomenon refers to sensitivity threshold issues at specific orientations relative to the static background magnetic field, either due to inefficient optical pumping or atomic polarization detection. Heading errors are systematic errors in DC field measurements across different orientations, caused by the nonlinear Zeeman effect [23,24,25], nuclear Zeeman effect [25,26], or light shifts [24]. Dead zones and heading errors essentially characterize the anisotropic response to quasi-static magnetic signals, aligning with the requirements of traditional applications like aeromagnetic surveys for geological anomalies. Substantial progress has been made in mitigating dead zones [27,28,29,30,31,32,33,34] and heading errors [2,23,24,25,26,33,34,35,36,37,38,39,40,41,42].

While prior research extensively investigated the orientation effects in quasi-static magnetic fields, such as dead zones and heading errors, emerging applications in unshielded biomagnetic research demand a new understanding of the orientation effects in dynamic magnetic signals. Unshielded biomagnetic detection allows for the detection of neuromagnetic and cardiomagnetic signals without the requirement of magnetically shielded rooms, potentially leading to a significant reduction in the cost of a biomagnetic system. While attempts at atomic-magnetometer-based magnetocardiography (MCG) in unshielded/low-shielded environments span decades [43,44,45,46,47,48], recent advances in sensor sensitivity and noise suppression techniques have enabled the detection of sub-pT brain neuromagnetic signals without magnetic shielding [49,50,51] and successful movable MCG recordings in unshielded settings [52]. Unlike quasi-static geomagnetic signals, biomagnetic signals measured in unshielded environments are high-frequency components (in the 1–1000 Hz range) [53] superimposed on a strong static background field. Such applications require atomic magnetometers to simultaneously fulfill two critical requirements: (1) the accurate characterization of amplitude–phase relationships across different frequencies in dynamic fields to prevent signal distortion, and (2) stable performance under different background field directions. In other words, the magnetometer requires an adequate frequency response across different orientations. This frequency response is defined as a function describing the amplitude scaling and phase shift characteristics of the magnetometer’s outputs when measuring alternating signals at varying frequencies. While the frequency response of atomic magnetometers at optimal orientations has been extensively examined [47,54,55,56,57,58,59,60,61,62], the dependence of frequency response on orientation is still not well understood. Achieving a better understanding of how orientation affects frequency response is vital for improving the accuracy of dynamic signal detection and for the development of magnetic noise cancellation techniques in unshielded conditions, such as active magnetic field stabilization and the magnetic gradiometers [50,59]. These advancements are essential for the development of practical wearable biomagnetic imaging systems operated in unshielded environments.

Here, we theoretically and experimentally study how the orientation of a Bell–Bloom atomic magnetometer influences its frequency response. With the help of the Bloch equation and employing the rotating wave approximation along with perturbative expansion, we derive the approximate analytical expression of the magnetometer’s frequency response under various orientations and verify it experimentally. We found that the frequency response of the magnetic measurement based on the demodulated quadrature component *Y* of magnetic resonance experienced orientation-related scaling, whereas the frequency response of the demodulated phase component θ was not affected by the magnetometer’s orientation. This orientation-independent feature is important for biomagnetic detection in magnetically unshielded environments where an orientation-robust measurement of AC magnetic signals is required.

## 2. Experiment Setup

The basic setup of the Bell–Bloom magnetometer used in this research is similar to that shown in Ref. [50]. We use a circularly polarized pumping light to polarize the atoms and use a linearly polarized light to monitor the evolution of the atomic spin. The pump beam is modulated around the Larmor frequency of the magnetometer to synchronize the atomic Larmor precession. The optical rotation of the probe beam is proportional to the overall magnetization along the propagation direction of the probe beam. The probe beam is set to a direction perpendicular to the pump beam, thereby reducing the scattered pump light that might be detected by the photodetector and minimizing interference. In our experiment, we used a 2 cm square atomic vapor cell filled with Cs, 300 Torr of helium gas, and 25 Torr of nitrogen gas. This vapor cell was kept at room temperature. Our pump light was locked to the transition from the ground state F=3 to the excited state $F^{\prime }=4$ on the Cs D1 line, corresponding to a wavelength of about 894.6 nm, which allows for the generation of spin polarization on the ground state F=4 via indirect optical pumping. This scheme is beneficial for reducing power broadening during optical pumping. The pump light was On–Off modulated with a peak power of 130 μW and a duty cycle of 2% by an acousto-optic modulator, with the modulation frequency set near the Larmor frequency. The frequency of the probe light was locked to the transition from the ground state F=3 to the excited state $F^{\prime }=4$ on the Cs D2 line, corresponding to a wavelength of about 852.3 nm, which enables the effective detection of spin evolution on the ground state F=4 via optical rotation while minimizing interference from spin evolution on F=3. The power of the probe light was 34 μW. The pump and probe beams are directed into the atomic cell through two separate fiber collimators, each with a 2.6 mm output diameter, ensuring they are perpendicular to each other and intersect. We detected the optical rotation of the probe beam using a polarimeter consisting of a polarizing beam splitter and a balanced photodetector. The resulting signal was demodulated by a lock-in amplifier to obtain the magnetic resonance signal. A more detailed description of the fundamental operating principles of this atomic magnetometer can be found in the supplementary materials in Ref. [50].

**Figure 1:**
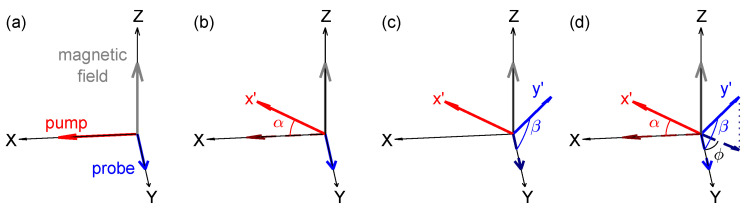
Orientation of the Bell–Bloom magnetometer. (**a**) The optimal orientation of a Bell–Bloom magnetometer with both pump and probe perpendicular to the magnetic field along the *z*-axis; (**b**) rotation of the pump beam by an angle α around the y-axis to align with the $x^{\prime }$ direction; (**c**) rotation of the probe beam by an angle β around the $x^{\prime }$-axis to align with the $y^{\prime }$ direction; (**d**) the final orientation of the Bell–Bloom magnetometer can be characterized with two angle α and β. The projection of $y^{\prime }$ onto the *x*-*o*-*y* plane forms an angle ϕ with the *y*-axis. This angle results in a phase shift of the demodulated magnetic resonance signal.

The optimal orientation for this type of magnetometer is one where both the pump and probe beams are perpendicular to the magnetic field, as shown in Figure 1a. This configuration results in the strongest magnetic resonance signal, thereby yielding the highest sensitivity. In practical applications, however, achieving this optimal condition may not always be feasible. For instance, in unshielded environments, the geomagnetic field is inclined with respect to the horizontal plane. To align the pump and probe beams perpendicularly to the ambient magnetic field, the magnetometer would need to be mounted on a specially designed fixture [51]. Nevertheless, mobile magnetic measurement inevitably deviates from the optimal orientation [52].

To conveniently parameterize the arbitrary orientation of the magnetometer, we consider rotating it from its optimal orientation (both the pump and probe beam perpendicular to the magnetic field) to any desired position, as shown in Figure 1. For the sake of theoretical convenience, we align the magnetic field along the *z*-axis. In the first step, the probe beam direction is kept stationary while the pump beam direction is rotated through an angle α to align with the $x^{\prime }$-axis, as shown in Figure 1b. Following this adjustment, the direction of the pump beam remains within the *x*-*o*-*z* plane. The angle α thus represents the deviation between the initial pump beam direction (along the *x*-axis) and the subsequent pump beam direction (along the $x^{\prime }$-axis). Subsequently, the direction of the pump beam was secured and the probe beam was rotated around the new direction of the pump (the $x^{\prime }$-axis) through an angle β, as shown in Figure 1c. As a result, the angle β now characterizes the deviation between the new probe beam direction, denoted as the $y^{\prime }$-axis, and the initial probe beam direction along the *y*-axis. Furthermore, the sensor can be rotated further around the *z*-axis to achieve any desired orientation. However, this rotation does not alter the angle between the magnetic field and either the pump or probe beams, thereby leaving the magnetic resonance signal unchanged. Consequently, in this paper, we restrict our analysis to the first two rotations, maintaining the pump beam within the *x*-*o*-*z* plane.

Under these conditions, the angles α and β are adequate to characterize the orientation of the magnetometer, as shown in Figure 1d, and the expressions for the unit direction vectors of the $x^{\prime }$- and $y^{\prime }$-axes are given by $\hat{e_{x^{\prime }}}=\left(\cos\alpha ,\;0,\;\sin\alpha \right)$ and $\hat{e_{y^{\prime }}}=\left(-\sin\alpha \sin\beta ,\;\cos\beta ,\;\cos\alpha \sin\beta \right)$, respectively. Throughout the rotation process, the pump and probe beams remain mutually perpendicular. This condition is readily maintained when both beams are confined within the sensor head of the magnetometer. We note that the projection of $y^{\prime }$ onto the *x*-*o*-*y* plane forms an angle ϕ with the *y*-axis. In the following analysis, we will show that this angle is very important for magnetic resonance, which is the angle that corresponds to its phase delay.

**Figure 2:**
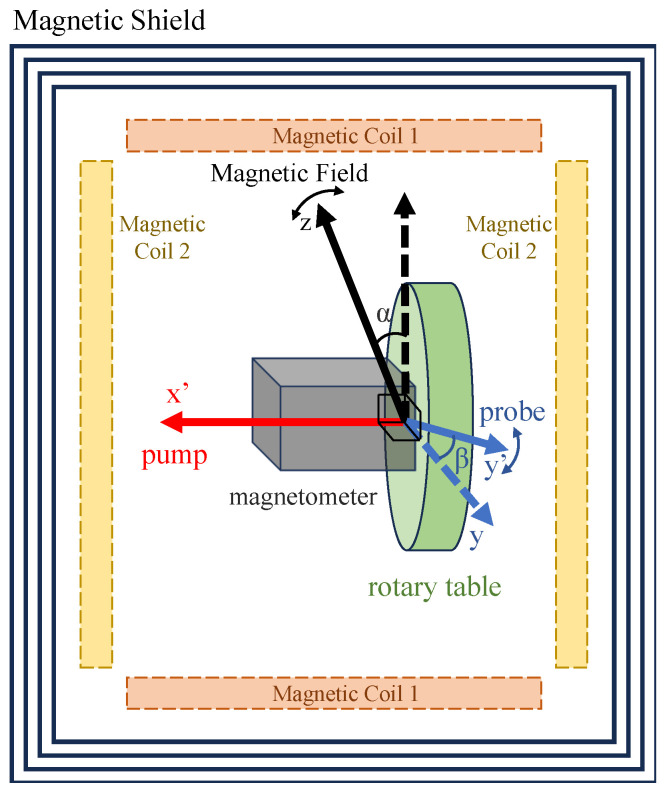
Experimental setup configuration. The magnetometer is affixed to a non-magnetic rotation stage, with the pump beam directed along the stage’s longitudinal axis and the probe beam aligned parallel to the stage’s horizontal plane. Two orthogonal coils generating uniform magnetic fields are employed to manipulate the background magnetic field. During the experiment, the direction of the pump beam is maintained constant, while the magnetic field direction is altered to effectuate a change in angle α. The rotation stage is utilized to rotate the probe beam around the pump beam, thus varying the angle β. The complete apparatus is housed within a multilayered magnetic shield.

The actual experimental setup is depicted in Figure 2, where we mounted the magnetometer on a non-magnetic rotation stage, and the entire apparatus was placed inside a multilayer magnetic shield. The pump beam was aligned along the axis of the rotation stage (the $x^{\prime }$-axis indicated by the red arrow in Figure 2) while the probe beam was parallel to the stage plane. We utilized two orthogonal uniform magnetic field coils to control the background magnetic field. Coil 1 generates a magnetic field along the vertical direction, as indicated by the black dashed arrow in Figure 2, while coil 2 produces a magnetic field horizontally along the $x^{\prime }$-axis. The optimal orientation for the magnetometer, where both the pump and probe beams are perpendicular to the magnetic field, is as follows: the pump beam is aligned horizontally along the $x^{\prime }$-axis; the magnetic field is oriented vertically, as indicated by the black dashed arrow; and the probe beam is directed along the direction perpendicular to the plane of the paper, as indicated by the blue dashed arrow marked for the *y*-axis.

In describing the arbitrary orientation of the magnetometer relative to the magnetic field, as shown in Figure 1, the involvement of two rotations is necessary. However, this is challenging to execute within the confined space of the magnetic shield. Therefore, in the actual experiment, we kept the direction of the pump beam constant. Instead of rotating the pump beam toward the magnetic field, we changed the currents in the two coils to rotate the magnetic field toward the pump beam by an angle α. Subsequently, we utilized the rotation stage to adjust the direction of the probe beam around the pump beam, thereby altering the β angle. The final directions for the magnetic field and the probe beam are along the *z*-axis and $y^{\prime }$-axis, respectively, as indicated by the black solid arrow and the blue solid arrow in Figure 2. In the experiment, the background magnetic field strength was maintained at approximately 1000 nT. The remanence inside the magnetic shield was less than 10 nT in all directions, as measured by a three-axis fluxgate sensor. No additional magnetic field was applied in the y-direction (perpendicular to the plane of the paper). Consequently, the strength and direction of the magnetic field were primarily controlled by the coils. The background magnetic field was directed within the plane parallel to the paper. For each specific experimental setup, the angle α between the background magnetic field and the vertical direction was adjusted to the desired value.

## 3. Theory

We use the Bloch equation to model the evolution of the atomic spin state, as shown in Equation (1):

(1) $$\begin{array}{c} \frac{dM}{dt}=-\gamma B\times M-\left(\Gamma_{2}M_{x}\hat{e_{x}}+\Gamma_{2}M_{y}\hat{e_{y}}+\Gamma_{1}M_{z}\hat{e_{z}}\right)-\Gamma_{P}\left(M-M_{0}\right), \end{array}$$

in which M is the magnetic moment of the atomic spin, and it is bold to indicate that it is a vector. The right side of Equation (1) describes the influence of three different effects on the evolution of M. The first part, −γB×M, describes the Larmor precession around the magnetic field B, in which γ is the magnetic ratio of ground state atomic spin and “×” indicates the cross product between two vectors. The second part, $-(\Gamma_{2}M_{x}\hat{e_{x}}+\Gamma_{2}M_{y}\hat{e_{y}}+\Gamma_{1}M_{z}\hat{e_{z}})$, describes the relaxation of the magnetization vector, which means that if there are no other effects, the transverse magnetization components $M_{x}\hat{e_{x}}$ and $M_{y}\hat{e_{y}}$ will decay exponentially to 0 with the transverse relaxation rate $\Gamma_{2}$, while the vertical magnetization components $M_{z}\hat{e_{z}}$ will decay exponentially to 0 with the vertical relaxation rate $\Gamma_{1}$. The third part, $-\Gamma_{P}\left(M-M_{0}\right)$, describes the optical pumping effect, in which $\Gamma_{P}$ is the pump rate that is proportional to the pump power and $M_{0}$ is the magnetization one can reach if there is no magnetic field and relaxation. The direction of $M_{0}$ is parallel to the propagation direction of the pump beam, and its amplitude is determined by the polarization of the pump beam. This part means that if there are no other effects, M will decay exponentially to $M_{0}$ with the pump rate $\Gamma_{P}$. Note that both B and $\Gamma_{P}$ can be time variant. The effect of the probe beam on M is mainly relaxation, which is already summarized in the relaxation part. The atomic magnetization will lead to an optical rotation of the probe beam, with an amplitude proportional to the projection of M on the direction of probe beam propagation, i.e., $M_{y^{\prime }}$.

As the magnetic field is along the *z*-axis in our setup, or $B=\left(0,0,B_{z}\right)$, Equation (1) can be simplified as


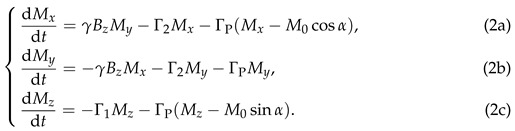


In our setup, the pump beam is square modulated at the angular frequency ω. Its Fourier expansion can be described as

(3) $$\begin{array}{c} \Gamma_{P}=\Gamma \cdot \sum_{n=-\infty }^{\infty }A_{n}e^{in\omega t}, \end{array}$$

in which $A_{n}$ is a complex constant describing the amplitude of the term with order *n* and i is the imaginary unit. By adjusting the phase of the modulation, we set $A_{1}$ to be a real number.

Following this, we calculate the expression of M. For $M_{z}$, we substitute Equation (3) into (2c). Considering that $\omega ∼\gamma B_{z}\gg \;\Gamma_{1}$, the contributions from the pump effect with n≠0 are fast oscillating and averaged to zero, so that in the steady state, the following occurs:

(4) $$\begin{array}{c} M_{z}\approx \frac{\Gamma A_{0}M_{0}\sin\alpha }{\Gamma_{1}+\Gamma A_{0}}. \end{array}$$

This result suggests that when the pump beam is perpendicular to the magnetic field B, e.g., angle α=0, $M_{z}$ is zero. As we rotate the pump beam around the *y*-axis, $M_{z}$ becomes larger and reaches a maximum when the pump beam is parallel with the magnetic field.

In order to calculate the expression for $M_{x}$ and $M_{y}$, we set $M_{+}=M_{x}+iM_{y}$ and further transfer it to the rotating frame as ${\tilde{M}}_{+}=M_{+}e^{-i\omega t}$. According to Equation (2a) and (2b), the evaluation of ${\tilde{M}}_{+}$ is

(5) $$\begin{array}{c} \frac{d{\tilde{M}}_{+}}{dt}=i\left(\gamma B_{z}-\omega \right){\tilde{M}}_{+}+\Gamma_{P}e^{-i\omega t}M_{0}\cos\alpha -\left(\Gamma_{2}+\Gamma_{P}\right){\tilde{M}}_{+}. \end{array}$$

Substituting Equation (3) into (5) and using rotating wave approximation to neglect the fast-oscillating terms, we have

(6) $$\begin{array}{c} \frac{d{\tilde{M}}_{+}}{dt}\approx i\left(\gamma B_{z}-\omega \right){\tilde{M}}_{+}+\Gamma A_{1}M_{0}\cos\alpha -\left(\Gamma_{2}+\Gamma A_{0}\right){\tilde{M}}_{+}. \end{array}$$

To calculate the frequency response of the magnetometer, we set $B_{z}=B_{0}+B_{s}\cos\left(\omega_{s}t\right)$, in which $B_{0}$ is a static magnetic field and $B_{s}\cos\left(\omega_{s}t\right)$ is an oscillating magnetic field with small amplitude serving as the magnetic signal to be measured. Typically, the angular frequency $\omega_{s}$ of this signal is much smaller than the $\gamma B_{0}$. Expanding ${\tilde{M}}_{+}$ as $\gamma B_{s}/\left(\Gamma_{2}+\Gamma A_{0}\right)$,

(7) $$\begin{array}{c} {\tilde{M}}_{+}=\sum_{m=0}^{\infty }{\tilde{M}}_{+}^{\left(m\right)}, \end{array}$$

the evolution of the *m*-th order is

(8) $$\begin{array}{cc} \frac{d{\tilde{M}}_{+}^{\left(m\right)}}{dt}\approx & i\left(\gamma B_{0}-\omega \right){\tilde{M}}_{+}^{\left(m\right)}+\left(1-\delta_{0,m}\right)i\gamma B_{s}\cos\left(\omega_{s}t\right){\tilde{M}}_{+}^{\left(m-1\right)}\\ & +\delta_{0,m}\Gamma A_{1}M_{0}\cos\alpha -\left(\Gamma_{2}+\Gamma A_{0}\right){\tilde{M}}_{+}^{\left(m\right)}, \end{array}$$

in which $\delta_{0,m}$ equals 1 when m=0 and 0 when m≠0. The steady states of ${\tilde{M}}_{+}^{\left(0\right)}$ is

(9) $$\begin{array}{c} {\tilde{M}}_{+}^{\left(0\right)}\approx \frac{\Gamma A_{1}M_{0}\cos\alpha }{i\left(\omega -\gamma B_{0}\right)+\left(\Gamma_{2}+\Gamma A_{0}\right)}, \end{array}$$

which describes a Lorentzian resonance known as magnetic resonance at $\omega =\gamma B_{0}$, e.g., when the modulation frequency of the pump beam is equal to the Larmor frequency of the static magnetic field $B_{0}$. The steady states of ${\tilde{M}}_{+}^{\left(1\right)}$ is

(10) $$\begin{array}{cc} {\tilde{M}}_{+}^{\left(1\right)}\approx & \frac{\gamma B_{s}}{2}\frac{\Gamma A_{1}M_{0}\cos\alpha }{\Xi }\\ & \times \left[\left(\frac{1}{\Xi -i\omega_{s}}-\frac{1}{\Xi +i\omega_{s}}\right)\cdot \sin\left(\omega_{s}t\right)+i\left(\frac{1}{\Xi -i\omega_{s}}+\frac{1}{\Xi +i\omega_{s}}\right)\cdot \cos\left(\omega_{s}t\right)\right], \end{array}$$

in which $\Xi =i\left(\omega -\gamma B_{0}\right)+\left(\Gamma_{2}+\Gamma A_{0}\right)$. This term describes the response to the magnetic signal $B_{s}\cos\left(\omega_{s}t\right)$.

Considering that $M_{+}=M_{x}+iM_{y}={\tilde{M}}_{+}e^{i\omega t}$ and that $M_{x}$ and $M_{y}$ are real numbers, we have Mx=Re(M˜+eiωt) and My=Im(M˜+eiωt), in which Re(c) and Im(c) represent the real part and imaginary part of the complex number *c*, respectively. As the probe signal Sig is proportional to the projection of the magnetic moment M on the direction of propagation of the probe beam $\hat{e_{y^{\prime }}}$, we have

(11) Sig=K·My′=K(M·ey′^)=K·(−Mxsinαsinβ+Mycosβ+Mzcosαsinβ)=K·[Im(M˜+(1−cos2αsin2β)eiωt−iϕ)+Mzcosαsinβ],

in which *K* represents a proportional coefficient and ϕ is an angle satisfying the conditions $\sin\phi =\sin\alpha \sin\beta /\sqrt{1-\cos^{2}\alpha \sin^{2}\beta }$ and $\cos\phi =\cos\beta /\sqrt{1-\cos^{2}\alpha \sin^{2}\beta }$. It means that the angle ϕ is the angle between the projection of the probe beam direction $y^{\prime }$ onto the *x*-*o*-*y* plane and the *y*-axis, as shown in Figure 1. According to Equation (4), (7), (9), and (10), the term Im(M˜+(1−cos2αsin2β)eiωt−iϕ) in Equation (11) describes an oscillation at the pump modulation frequency ω, in which a phase delay of −ϕ is introduced, and the term $M_{z}\cos\alpha \sin\beta$ is a steady offset. As we used a lock-in amplifier to demodulate the probe signal Sig at the modulation frequency ω, we can express Sig as follows to obtain the demodulated results:

(12) $$\begin{array}{cc} Sig & =X\sin\left(\omega t\right)+Y\cos\left(\omega t\right)+K\cdot M_{z}\cos\alpha \sin\beta \\ \; & =R\sin\left(\omega t+\theta \right)+K\cdot M_{z}\cos\alpha \sin\beta , \end{array}$$

in which *X*, *Y*, *R*, and θ are real numbers representing the demodulated in-phase signal, quadrature signal, magnitude signal, and phase signal, respectively. According to Equation (11) and (12), the specific forms of these demodulated signals are as follows:


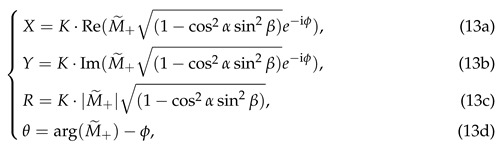
in which |c| and arg(c) represent the magnitude and phase angle of the complex number *c*, respectively.

For the case where the background magnetic field is steady, i.e., the magnetic modulation amplitude $B_{s}$ vanishes and the z-component of the magnetic field $B_{z}$ equals the constant $B_{0}$, we have ${\tilde{M}}_{+}{|}_{B_{s}=0}={\tilde{M}}_{+}^{\left(0\right)}$. As a result, according to Equation (9) and (13), the demodulated results are as follows:


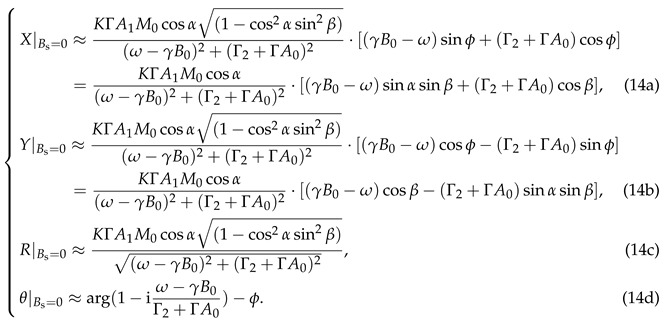


**Figure 3:**
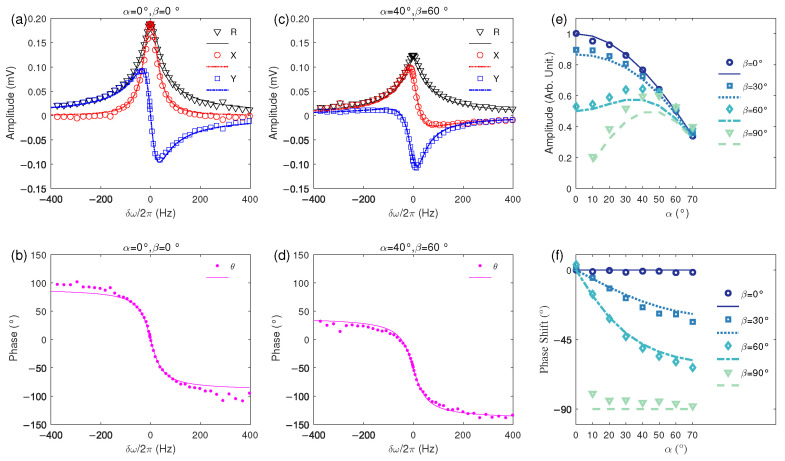
Magnetic resonances with different orientations of the Bell–Bloom magnetometer. (**a**) The demodulated *R*, *X*, and *Y* and (**b**) phase angle θ of the magnetic resonance signal with $\alpha =0^{\circ },\beta =0^{\circ }$, with the horizontal axis being the detuning of the pump modulation, i.e., $\delta \omega =\omega -\gamma B_{0}$; (**c**) the demodulated R, X, and Y and (**d**) θ of the magnetic resonance signal with $\alpha =40^{\circ },\beta =60^{\circ }$; (**e**) the amplitudes and (**f**) phase shifts of magnetic resonances with different orientations. The dots in (**a**–**d**) are the experimentally recorded demodulation signals, and the lines in (**a**–**d**) are the Lorentzian fitting results. The dots in (**e**,**f**) are the normalized Lorentzian fitted amplitudes and phase shifts of magnetic resonances, and the lines are calculated results according to Equation (14).

Equation (14) exhibits clear magnetic resonance characteristics, such as *R* reaching its maximum value under the resonance condition where the modulation frequency of the pump beam equals the Larmor frequency (i.e., $\omega =\gamma B_{0}$). Hence, the waveforms of these demodulated results with respect to the pump modulation detuning $\delta \omega =\omega -\gamma B_{0}$ are referred to as magnetic resonance curves, which exhibit a Lorentzian lineshape. A change in the orientation of the magnetometer affects the amplitude of *R*, which is proportional to $\cos\alpha \sqrt{1-\cos^{2}\alpha \sin^{2}\beta }$. In addition to amplitude variations, an overall phase shift is introduced in the magnetic resonance, which alters the waveforms of *X* and *Y* with respect to the detuning δω. The shape of θ remains unchanged, experiencing only an overall phase shift of −ϕ. This characteristic helps to avoid the influence of the magnetometer’s orientation on its frequency response. The influence of the magnetometer orientation on these magnetic resonance curves will be illustrated in more detail in Figure 3.

When a modulated magnetic field $B_{s}\cos\left(\omega_{s}t\right)$ is superimposed on the background field, i.e., $B_{z}=B_{0}+B_{s}\cos\left(\omega_{s}t\right)$, AC components with frequency $\omega_{s}$ and an amplitude proportional to $B_{s}$ will emerge in the lock-in amplifier demodulated signals *Y* and θ. This phenomenon originates from the contribution of ${\tilde{M}}_{+}^{\left(1\right)}$ as described in Equation (10). These AC components, after calibration, can subsequently serve as measurement values for the modulated magnetic field. Their amplitude and phase responses to modulated magnetic fields of various frequencies represent the frequency response of the magnetometer. Specifically, for calculating the amplitude–frequency response at the resonance condition $\omega =\gamma B_{0}$, the amplitude scaling factor is obtained by normalizing the AC component amplitudes of the *Y* or θ signals measured under specific α, β, and $\omega_{s}$ against those under the optimal orientation ($\alpha =\beta =0^{\circ }$) and quasi-static limit ($\omega_{s}\to 0$ Hz). For the phase–frequency response, the phase shift is derived by subtracting the phase of the magnetic field modulation from the phase of the AC component in the *Y* or θ signals. Based on Equations (7), (9), (10), and (13), the frequency responses corresponding to *Y*-readout and θ-readout are expressed, respectively, as follows:


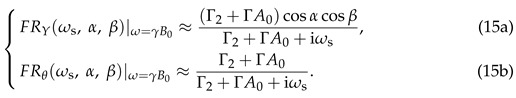


The frequency response is represented by a complex number associated with the magnetic modulation frequency $\omega_{s}$. The magnitude and phase of this complex number, respectively, represent the amplitude scaling and phase shift of the signal with frequency $\omega_{s}$ in the magnetometer readout. As shown in Equation (15), the frequency responses of both the *Y*-readout and the θ-readout are those of a first-order Butterworth low-pass filter with a bandwidth of $\Gamma_{2}+\Gamma A_{0}$, in which $\Gamma_{2}$ and $\Gamma A_{0}$ represent the transverse relaxation rate and the power broadening induced by the pump light, respectively. By comparing Equation (14) with (15), it can be observed that the bandwidth of the frequency response equals the linewidth of the magnetic resonance. When the magnetometer is oriented in different directions, the frequency response of the *Y*-readout varies proportionally to cosαcosβ, noting that this variation differs from the $\cos\alpha \sqrt{1-\cos^{2}\alpha \sin^{2}\beta }$ scaling law governing the *R*-magnitude. In contrast, the frequency response of the θ-readout shows no direct relationship with the magnetometer’s orientation angles α and β. The direction-independent frequency response characteristic of the θ-readout provides an opportunity to mitigate the influence of magnetometer orientation on frequency response.

## 4. Results

We first examine the influence of magnetometer orientation on the amplitude and phase shift of the magnetic resonance signal. To do so, we set the strength of the background magnetic field to be about 1000 nT and adjusted the values of angle α ranging from 0 to 70 degrees and β from 0 to 90 degrees. At each orientation, we scanned the modulation frequency of the pump beam within a range of ±400 Hz around the Larmor frequency and recorded the corresponding demodulated values of *R*, *X*, *Y*, and θ, thereby obtaining the magnetic resonance signals. Examples of magnetic resonance with $\alpha =0^{\circ },\beta =0^{\circ }$ and $\alpha =40^{\circ },\beta =60^{\circ }$ are shown in Figure 3a,b and c,d, respectively. The dots there are the experimentally recorded demodulation signals and the lines are the Lorentzian fitting results. From the figure, we can observe that when $\alpha =0^{\circ },\beta =0^{\circ }$, the demodulated *R* and *X* exhibit even symmetry with respect to the resonance frequency, while *Y* and θ show odd symmetry with respect to the resonance frequency. When $\alpha =40^{\circ },\beta =60^{\circ }$, the value *R* remains evenly symmetric with respect to the resonance frequency, but its amplitude decreases; the shapes of *X* and *Y* undergo significant distortion; and the shape of θ does not change, but there is an overall phase shift downward.

We summarize the influence of the magnetometer orientation on these amplitude variations and phase shifts in Figure 3e,f. The dots there are the fitted amplitudes and phase shifts, and the lines are the calculated results according to Equation (14), i.e., the amplitude scaling is $\cos\alpha \sqrt{1-\cos^{2}\alpha \sin^{2}\beta }$ and the phase shift is −ϕ=−arg(cosβ+isinαsinβ). From Figure 3e, we can observe that, in general, the configuration with $\alpha =0^{\circ },\beta =0^{\circ }$ exhibits the maximum amplitude, while other combinations of α and β result in smaller signal amplitudes, which is consistent with the theory. Furthermore, we note that when $\beta =30^{\circ }$ and $\beta =60^{\circ }$, the signal amplitude initially increases and then decreases as α increases. This is due to the interplay of two opposing trends: as α increases, the effective pumping efficiency decreases, leading to a reduction in the oscillating component of the magnetic moment, which in turn decreases the signal amplitude; simultaneously, as α increases, the projection of the probe beam onto the direction perpendicular to the magnetic field increases, making the detection process more effective. Furthermore, it can be seen that for the cases where $\beta =30^{\circ }$ and $\beta =60^{\circ }$, the actual measured magnetic resonance amplitudes are larger than the theoretical values. This could be due to the fact that the Bloch equations we used do not take into account the specific pumping and relaxation rates at the nine magnetic sublevels of the F=4 ground state. In reality, the F=4 magnetic resonance signal is composed of a combination of eight different magnetic resonance peaks, which have the same resonance frequency but differ in amplitude and slightly in linewidth [59]. As the direction of the magnetic field changes, the relative amplitudes between these sub-magnetic resonance peaks also change. A more detailed investigation using density matrix equations might lead to a better agreement between theory and experiment [63]. In contrast, as shown in Figure 3f, the phase shift is in better agreement with the theoretical predictions, which might be due to the fact that the phase shift directly depends on α and β and is less affected by the magnetic resonance amplitude and linewidth. We can observe that when $\beta =0^{\circ }$ or $\beta =90^{\circ }$, the change in α does not lead to a significant phase shift. However, when β is between $0^{\circ }$ and $90^{\circ }$, the phase deviation increases with increasing α, trending toward −β as α approaches $90^{\circ }$.

**Figure 4:**
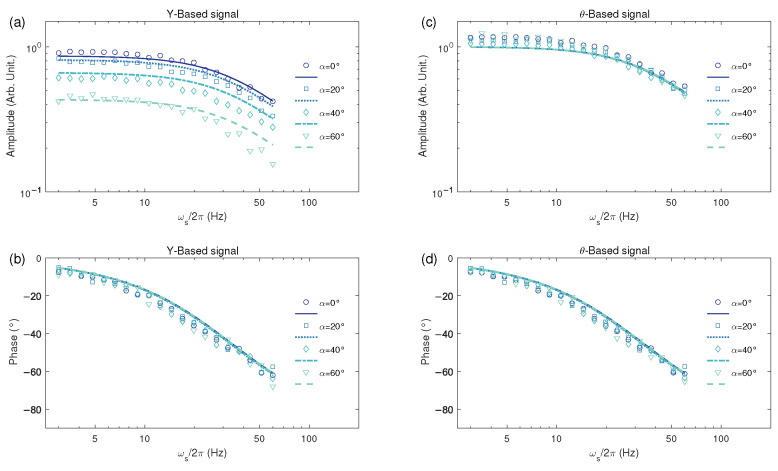
Frequency response at different orientations, with α ranging from $0^{\circ }$ to $60^{\circ }$, $\beta =30^{\circ }$, and $\omega =\gamma B_{0}$. (**a**) Amplitude–frequency response and (**b**) phase–frequency response of magnetic measurement based on *Y*-readout; (**c**) amplitude–frequency response and (**d**) phase–frequency response of magnetic measurement based on θ-readout. The dots are experimentally measured frequency responses and the lines are calculated results according to Equation (15).

We also check the influence of magnetometer orientation on its frequency response. The frequency responses with α ranging from $0^{\circ }$ to $60^{\circ }$, $\beta =30^{\circ }$, and $\omega =\gamma B_{0}$ are shown in Figure 4, where the dots are experimentally measured frequency responses and the lines are calculated results according to Equation (15). The subplots in the left and right columns of Figure 4 represent the frequency responses of the *Y*-readout and θ-readout, respectively. The subplots in the first and second rows represent the amplitude–frequency responses and the phase–frequency responses, respectively.

We measured and processed the data as follows. First, we measured the magnetic resonance signals under different orientations and utilized the fitted magnetic resonance linewidths to calculate the expected frequency response according to Equation (15). For each orientation, we kept the strength of the background magnetic field at about 1000 nT and adjusted the orientation angles α and β to the desired values. Then, similar to the situation depicted in Figure 3, we scanned the pump modulation frequency within a range of ±400 Hz around the Larmor frequency and acquired the magnetic resonance signals. We performed a Lorentzian fit on the magnetic resonance signals to obtain the linewidth, amplitude, phase shift, and center frequency. According to Equation (15), and combining the fitted magnetic resonance linewidth (which is about 2π×33 Hz and is the same as the bandwidth of the frequency response), we plotted the expected frequency responses shown as lines in Figure 4.

Then, we measured the experimental frequency response of the magnetometer. The pump beam modulation frequency was first set to resonance condition $\omega =\gamma B_{0}$. A supplementary cosine-modulated magnetic field $B_{s}\cos\omega_{s}t$ with $B_{s}=1$ nT was then applied along the background field direction. This modulation induces AC signals at $\omega_{s}$ in both *Y*- and θ-readouts. The normalized amplitude scaling coefficients were obtained by dividing these signal amplitudes by the reference amplitudes, which were derived under optimal orientation ($\alpha =\beta =0^{\circ }$) and quasi-static modulation ($\omega_{s}\to 0$ Hz). Specifically, the reference amplitudes were estimated through $Ref_{Y}=\left(-dY/d\omega {|}_{B_{s}=0,\;\omega =\gamma B_{0},\;\alpha =\beta =0^{\circ }}\right)\cdot \gamma B_{s}=K\Gamma A_{1}M_{0}/{\left(\Gamma_{2}+\Gamma A_{0}\right)}^{2}$ and $Ref_{\theta }=\left(-d\theta /d\omega {|}_{B_{s}=0,\;\omega =\gamma B_{0},\;\alpha =\beta =0^{\circ }}\right)\cdot \gamma B_{s}=180^{\circ }/\left[\pi \left(\Gamma_{2}+\Gamma A_{0}\right)\right]$, which can be calculated from the magnetic resonance fitting results with $\alpha =\beta =0^{\circ }$. Phase shifts were determined by measuring the relative phase difference between the readout signals and applied magnetic modulation. The magnetic modulation frequency $\omega_{s}$ scans from 3 Hz to 60 Hz yielded the amplitude–frequency and phase–frequency responses for both readout channels, as shown by the experimental data points in Figure 4.

As demonstrated in Figure 4, both *Y*- and θ-readouts exhibit frequency responses consistent with first-order Butterworth low-pass filter characteristics. The amplitude response decreases progressively with increasing magnetic modulation frequency, accompanied by corresponding phase accumulation. While deviations from the optimal orientation ($\alpha =\beta =0^{\circ }$) induce overall downward shifts in the *Y*-readout’s amplitude–frequency response (Figure 4a), its phase–frequency response remains unaffected (Figure 4b). Notably, although the magnetic resonance lineshape of *Y* experiences the orientation-dependent distortion described by Equation (14b), counterintuitively, the frequency response of *Y*-readout maintains the canonical Butterworth profile without deformation (Equation (15a)). In contrast, both amplitude– and phase–frequency responses of the θ-readout show orientation invariance (Figure 4c,d).

The orientation-related amplitude scaling of *Y*-readout introduces significant operational constraints. When using Y-readouts, calibrated at a fixed direction, for AC field measurements, directional misalignment causes signal attenuation proportional to cosαcosβ, leading to the systematic underestimation of true field amplitudes. If the *Y*-readout is used as input to control certain closed-loop feedback processes, such as the closed-loop operation of the atomic magnetometer [55,64] or active magnetic field stabilization [65,66,67], this variable gain modifies the loop transfer function, altering key performance parameters including bandwidth, overshoot, and potentially inducing instability.

The θ-readout’s orientation-independent frequency response eliminates these issues, enabling accurate AC field measurements and stable feedback control. However, Equation (14d) reveals an orientation-dependent phase bias −ϕ in the θ-readout, manifesting as heading errors. This artifact originates from non-parallel pump–probe propagation, with the phase offset given by −ϕ=−arg(cosβ+isinαsinβ). Compensation strategies include real-time phase-bias correction through orientation monitoring or co-aligned pump–probe beam configurations. Parallel/antiparallel beam alignment eliminates orientation-related phase bias but typically results in the detection of pump light, thereby affecting the measurement; however, this can be resolved with high-quality optical filters. Additionally, there is a potential benefit: if the diffusion optical pumping scheme [68] is adopted, this can suppress the undesirable light shifts and power-broadening associated with optical pumping.

## 5. Conclusions and Discussion

In this study, we investigated how the orientation of the Bell–Bloom magnetometer relative to the background magnetic field influences its frequency response. This research was motivated by the critical need to address orientation-induced inaccuracies in AC signal detection for magnetically unshielded biomagnetic measurements—an emerging field that promises to eliminate costly magnetic shields, dramatically reduce system costs, and accelerate the translation of biomagnetic technologies from laboratory research to clinical applications [22,50,51]. By utilizing the Bloch equation, the rotating wave approximation, and perturbative expansion, we derived an approximate analytical expression for the magnetometer’s frequency response under different orientations. Using a homemade Bell–Bloom Cs magnetometer with a perpendicular pump and probe lights, we compared the frequency responses of magnetic measurements based on the demodulated quadrature component (*Y*) and the demodulated phase component (θ) of magnetic resonance.

We observed that although the magnetic resonance lineshape of *Y* deforms with changes in the magnetometer’s orientation, the frequency response of the *Y*-readout maintains the shape of a first-order Butterworth low-pass filter with a bandwidth around the magnetic resonance linewidth. However, as the magnetometer’s orientation deviates from the optimal direction (where both pump and probe lights are perpendicular to the background magnetic field), the amplitude–frequency response of the *Y*-readout decreases. This decline leads to several issues, such as the systematic underestimation of true field amplitudes when *Y*-readout is directly used for AC field measurement and the alteration of key performance parameters in closed-loop implementations, including the closed-loop operation of the magnetometer [55,64] or active magnetic field stabilization [65,66,67].

In contrast, the frequency response of the θ-readout, which also resembles a first-order Butterworth low-pass filter with a bandwidth around the magnetic resonance linewidth, does not vary with changes in the magnetometer’s orientation. This characteristic is beneficial for accurate AC field measurements and stable feedback control, which is particularly important for magnetically unshielded biomagnetic detection where the orientation of the magnetometer may have randomness and the detection signal contains significant high-frequency components. However, the θ-readout exhibits a phase offset related to the magnetometer’s orientation, which can cause heading errors. Since this phase offset arises from the non-parallelism of the pump and probe lights in the magnetometer and depends solely on the magnetometer’s orientation, it can be compensated by continuously monitoring the magnetometer’s orientation or by adopting a parallel or antiparallel pump–probe configuration. The potential advantage of such a configuration is the utilization of a diffusion optical pumping scheme [68] to eliminate light shifts and power broadening induced by the pump light in the magnetic resonance spectrum.

Our research is important for biomagnetic detection in magnetically unshielded environments where orientation-robust measurements of AC magnetic signals are required. Biological magnetic signals exhibit high-frequency characteristics distinct from traditional geomagnetic detection, and wearable devices may require the magnetometer to face different orientations relative to the background field. Therefore, understanding the magnetometer’s frequency response across orientations is critical to optimize the detection accuracy of alternating biomagnetic signals. Our findings indicate that using a phase component θ instead of a quadrature component *Y* mitigates orientation-dependent variations in frequency responses. Though the existing literature demonstrates the coexistence of both approaches, for instance, Refs. [34,59,60,69] used *Y*-readout while Refs. [47,62] chose θ-readout, previous studies might have selected the θ-readout for other considerations (such as noise performance, as mentioned in Ref. [47]). Our results establish θ-readout superiority specifically for orientation-robust measurements of AC magnetic signals—a critical factor in unshielded biomedical applications.

For wearable biomagnetic detection under magnetically unshielded conditions, such as human brain neuromagnetic signals recording or MCG, there are still some further improvements to be implemented. Currently, as a fundamental mechanistic study, our research has only utilized a 1000 nT background magnetic field in a magnetic shield. In subsequent experiments under the unshielded environment with a larger background field, for example, 50 μT, the influence of the nonlinear Zeeman effect on the frequency response needs to be considered [59]. Furthermore, to achieve the required sensitivity for brain and heart magnetic field detection, more systematic optimization of experimental parameters such as laser power and vapor cell temperature is necessary for future research. During this process, new factors influencing frequency response may be identified. For example, when the atomic vapor cell is heated to enhance the magnetometer’s sensitivity, the enhanced light–atom interaction may induce orientation-related light absorption and power broadening [34], which in turn influence the frequency response.

## Data Availability

All data needed to evaluate the conclusions in the paper are present in the paper. Additional data related to this paper may be requested from the authors.
